# Percutaneous injection of ethanol for thyroid nodule treatment: a comparative study

**DOI:** 10.20945/2359-3997000000363

**Published:** 2021-04-29

**Authors:** Daysi Maria de Alcântara-Jones, Lucas Moura Bastos Borges, Tania Freitas A. Nunes, Gabriella Brandão Pita, Vinicius Brito Rocha, Julia Mandaro Lavinas, Leila Maria Batista Araújo, Luis Fernando Fernandes Adan

**Affiliations:** 1 Universidade Federal da Bahia Hospital São Rafael Faculdade de Medicina da Bahia Salvador BA Brasil Faculdade de Medicina da Bahia, Universidade Federal da Bahia (UFBA), Hospital São Rafael (HSR), Salvador, BA, Brasil.; 2 Universidade Federal da Bahia Faculdade de Medicina Salvador BA Brasil Faculdade de Medicina da Bahia, Universidade Federal da Bahia (UFBA), Salvador, BA, Brasil.; 3 Hospital Santo Antonio Pós-Graduação em Medicina Salvador BA Brasil Pós-Graduação em Medicina, Hospital Santo Antonio, Salvador, BA, Brasil.

**Keywords:** Thyroid nodule, nodular goiter, ethanol, ablation techniques

## Abstract

**Objective:**

Percutaneous ethanol injection (PEI) is an alternative to surgery for the treatment of thyroid nodules (TNs). However, size reductions of treated (TTNs) and untreated TN (UTNs) have not been compared. Volumetric reductions in TTNs with PEI were evaluated by comparing TTNs and UTNs in the same patient, and independent variables predicting good post-PEI outcomes were analyzed.

**Materials and methods:**

Overall, 282 patients with multinodular goiters were selected. Two nodules located in different lobes were compared for common disease behaviors. Overall, 150 nodules were selected from 75 patients (6 M: 69 F) with a mean age of 50.1 ± 17.4 years. This prospective nonrandomized intervention study prioritized treating TNs of greater volume or single hyperfunctioning TNs. A single observer experienced in PEI and an ultrasound specialist performed the interventions.

**Results and discussion:**

TTNs (mean volume: 14.8 ± 16.2 mL) were reduced by 72.6 ± 27.3% of their initial volume, while UTNs increased by a mean of 365.7 ± 1.403.8% (p < 0.00001). The patients underwent a mean of 4.0 ± 3.1 outpatient PEI sessions without relevant complications. Logistic regression analysis showed that the magnitude of the PEI induced reduction was associated with the number of treatment sessions (p = 0.03, CI [1.1-38.2]) and not with ultrasonographic characteristics of the nodules. Each PEI session increased the rate of TN reduction by a factor of 6.7.

**Conclusions:**

PEI is a well-tolerated outpatient procedure that effectively reduces the volume of TNs and is noticeably superior to conservative treatment for all ultrasonographic classifications.

## INTRODUCTION

Many studies have reported a high frequency of thyroid nodules (TNs) in autopsies of people without thyroid dysfunction (1–3). More than 50% of women aged over 50 years have a TN (2). More than 90% of TNs are benign (3), and few will grow to compress cervical structures, causing esthetic concerns or hyperfunctioning nodules. Thus, the ideal treatment for such a prevalent pathology should be easy to perform, free of complications, and cost effective.

Benign TNs are managed through observation, leading to good outcomes (4). Surgical TN treatment, the main therapeutic option for malignant TNs and large benign TNs, is cautiously considered, even in certain malignant TN cases (5,6). Surgery often results in definitive hypothyroidism, and rarely, recurrent laryngeal nerve lesions and hypoparathyroidism are reported to produce dysphonia and hypocalcemia associated with changes in bone quality (7). Sclerotherapy by percutaneous ethanol injection (PEI), endorsed as a safe and effective alternative for cystic TNs (8,9), is also an effective therapy to reduce solid TNs of different sizes (10), whether hyperfunctioning or not. PEI is a safe outpatient procedure without complications and with short- and long-term TN-reducing effects. Thus, the objective of the present study is to evaluate PEI for multinodular goiter treatment.

## MATERIALS AND METHODS

This prospective nonrandomized intervention study used convenience sampling and a nonrandomized control group due to ethical considerations. The largest (dominant) or hyperfunctioning nodule was treated to respect this clinical interest.

The multinodular goiter patients had a PEI-treated (case) and a PEI-untreated (control) nodule. These were in different lobes to avoid confounding treated and untreated TNs. A volume reduction below 0.7% between sessions represented no reduction (10).

In total, 282 patients were treated using PEI between May 2001 and August 2017, initially at Professor Edgar Santos University Hospital and subsequently at São Rafael Hospital (Salvador, BA, Brazil). Of these initial 282, 75 patients with multinodular goiter (two TNs) were selected, totaling 150 nodules evaluated. The inclusion criteria were older than 16 years of age, nodules diagnosed as benign by two puncture aspirations, and no family history of thyroid cancer. Thyroid hormone treatment for the sole purpose of reducing TN volume, a protocol no longer recommended by the American Thyroid Association (8), was discontinued before alcohol sclerotherapy.

The patients underwent clinical and laboratory evaluation, including 99mTechnetium-sestamibi thyroid scintigraphy and a radioactive iodine uptake test (provided by *Instituto de Pesquisas Energéticas e Nucleares* – IPEN, São Paulo, Brazil), when excess thyroid hormone and absent antithyroid antibodies suggested a hyperfunctioning TN. Patients with a heterogeneous pattern on thyroid ultrasound, characteristic of thyroiditis, were excluded to avoid confounding TN and thyroiditis pseudonodules.

The ALOKA SSD 1700 software DYNAVIEW II® Doppler ultrasound scanner with a 7.5-MHz transducer was used for the ultrasonographic study to classify the TNs as follows: 1) solid, 2) predominantly solid with cysts, 3) mixed, when it was not possible to measure cysts dispersed in solid areas, 4) predominantly cystic, and 5) cystic.

Both treated (TTNs) and untreated (UTNs) TNs were measured at their largest diameter, and their volume was calculated using height, width, and anteroposterior diameter multiplied by a constant (0.52). Absolute (99.6 GL) and sterile ethanol (v/v) (both ethanol products from Health Tech, Alto da Mooca, São Paulo, Brazil) were added to 10 mL ampoules using 5 mL and 10 mL disposable syringes. The ethanol dose injected percutaneously during each PEI session was not previously defined, as it was based on the level of patient acceptance and specific TN characteristics. In cystic lesions, the volume of ethanol injected was approximately the same as the volume of liquid content aspirated. The procedure was performed by the same professional (Daysi Alcântara-Jones) experienced with this technique since 2001.

The degree of TN volume reduction was calculated using the equation: $Degree\;of\;reduction\;\left(\%\right)=100-\left(\frac{100\;x\;Vf}{Vi}\right)$, where *Vf* is the final volume (after treatment) and *Vi* is the initial volume (before treatment). The degree of TN reduction in the largest diameter was calculated by $Degree\;of\;reduction\;\left(\%\right)=100-\left(\frac{100\;x\;Mf}{Mi}\right)$, where *Mf* is the largest diameter of the nodule after PEI and *Mi* is the largest diameter before PEI. The TNs that grew were registered with negative values to indicate a negative reduction. To understand the relationship between the number of PEI sessions and TN reduction, the variable “number of sessions” was stratified into “one session”, “two to four sessions” and “five sessions or more”.

The criteria for evaluating completion of treatment were as follows: nodule size smaller than 0.5 cm^3^ or 1.3 cm in its largest diameter; increased consistency, which made ethanol injection difficult; and absence of vascularization in a previously solid TN under treatment. The treatment was also discontinued when the needle was obstructed in solid nodules with calcification foci.

### Statistical analysis

Demographic data are expressed as the mean, median, and standard deviation. Thyroid-stimulating hormone (TSH) levels were stratified as hyperthyroidism, subclinical hyperthyroidism, and euthyroidism. The normality of the measurement distributions was assessed using the Shapiro-Wilk test. The paired t-test was used to compare the volume, largest diameter, and degree of reduction in TTNs and UTNs, and the Wilcoxon test was used to compare nonparametric variables. The Kruskal-Wallis test was used to assess the degree of TTN reduction by ultrasound classification. The statistical software R version 3.6.3 was used for logistic regression analysis, which assessed which independent variables influenced TN reduction. A p-value < 0.05 was considered significant for all analyses. All volunteers signed the informed consent form after full explanation of the purpose and nature of all procedures used. The authors ensured that research involving human subjects complied with the Declaration of Helsinki, and the study was approved by the Research Ethics Committee (REC) of São Rafael and of Edgard Santos University Hospital (Salvador, BA, Brazil) in 2003, which was updated in the Plataforma Brasil database in 2017 (registration number: 2.597.674).

## RESULTS

One hundred fifty patients (mean age, 50.1 ± 17.4; median, 46.5; 92.0%, female), with ages ranging between 16 and 84 years, participated in the study. No hypothyroidism cases were diagnosed during selection or after treatment (TSH mean ± SD (μIU/mL): 1.3 ± 1.1, T4_L_ mean ± SD (ng/dL): 1.3 ± 1.0). There were 12 hyperfunctioning TNs: 4 (6.5%) hyperthyroidism and 8 (13.1%) subclinical hyperthyroidism. Ultrasonographic standards of TTNs were as follows: solid: 29 (39.1%), predominantly solid: 12 (16.2%), predominantly cystic: 11 (14.9%), cystic: 9 (12.2%), and mixed: 13 (17.5%). Ultrasonographic standards of UTNs were as follows: solid: 32 (47.0%), predominantly solid: 10 (14.7%), predominantly cystic: 4 (5.9%), cystic: 13 (19.1%), and mixed: 9 (13.2%). There was loss of information on the ultrasound pattern of one TTN and seven UTNs. TTN localization was as follows: right side: 44 (58.7%), left side: 30 (40.0%), and isthmus: 1 (1.3%). UTN localization was as follows: right side: 33 (44.0%), left side: 35 (46.7%), and isthmus: 7 (9.3%).

**Figure 1:**
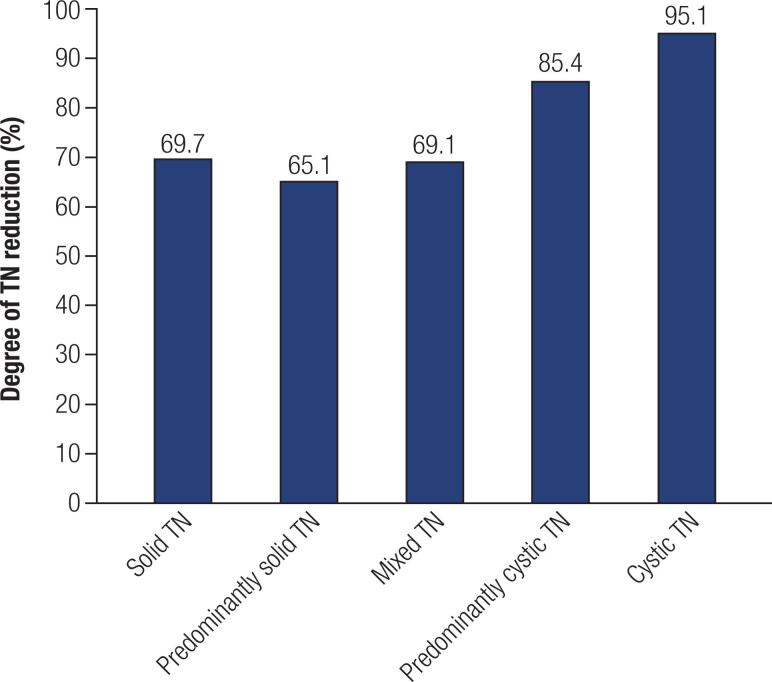
Degrees of volume reduction in TTNs by ultrasonographic standard. The degrees of largest diameter reduction in the TTNs by ultrasonographic standards were as follows: (solid TN: 40.7%, predominantly solid TN: 34.8%, mixed TN: 35.2%, predominantly cystic TN: 53.1%, cystic TN: 63.8%).

Table 1 shows that the difference between the measurements of the TTNs before and after PEI was highly significant with regard to TNs volume and largest diameter, while the table shows that there were no significant reductions in UTNs with regard to TNs volume and largest diameter. The difference in the degree of reduction in the volume of the TTNs (72.6 ± 27.3% [mean ± SD]) and UTNs (-365.7 ± 1403.8% [mean ± SD]) was very significant (p-value: 5.0 x 10^-12^) and similar to the degree of reduction in the largest diameter of TTNs (43.2 ± 28.3 [mean ± SD]) compared to UTNs (-85.4 ± 251.6 [mean ± SD]) (p-value: 1.1 x 10^-12^). The UTNs, unlike the TTNs, grew during the observation period but developed no clinical signs due to their small size (mean initial largest diameter of 1.1 cm ± 0.8 SD and mean initial volume of 0.7 ± 1.4 SD mL). The percentages of the mean reduction in TTNs distributed by each ultrasound standard are shown in Figure 1.

**Table 1:** Metric indices of untreated and treated TNs using PEI

	Before	After	P-value
**Treated TN**			
Volume in cm3 (mean ± SD) (N = 75)	14.8 ± 16.2	3.5 ± 5.5	2.5 × 10^-8^
Largest diameter in cm (mean ± SD) (N = 75)	3.9 ± 2.1	2.1 ± 1.2	10.8 × 10^-8^
**Untreated TN**			
Volume in cm3 (mean ± SD) (N = 71)	0.73 ± 1.4	1.3 ± 2.6	0.32
Largest diameter in cm (mean ± SD) (N = 73)	1.1 ± 0.8	1.4 ± 1.0	0.3

Abbreviations: PEI, percutaneous ethanol injection; TN, thyroid nodule

**Figure 2:**
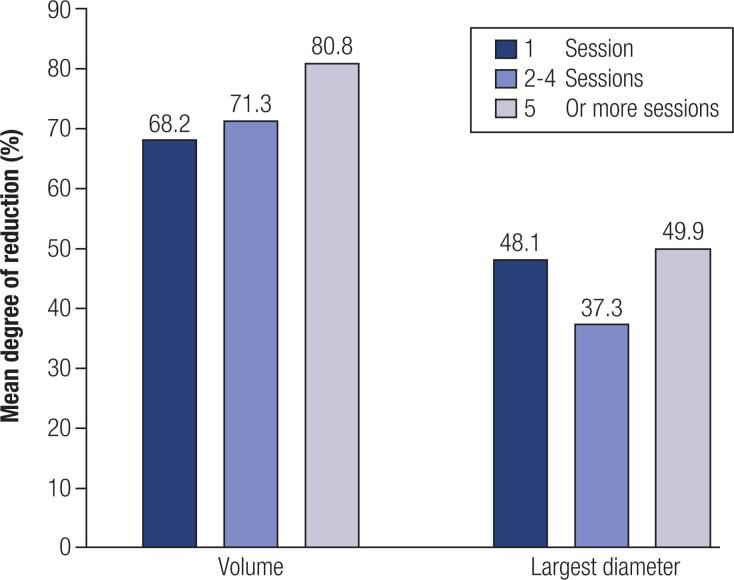
Degree of reduction in TTNs stratified by number of sessions.

The number of sessions was heterogeneous, ranging from one to eight sessions. The patients underwent 4.0 ± 3.0 (median, 3) PEI sessions, and the duration of treatment was 22.6 ± 27.6 (median, 10) months. Figure 2 shows the mean reduction percentage achieved in TTNs stratified by the number of sessions. Only 43 (57.3%) patients met the criteria to complete the treatment and were observed in the long term. The results of the other 32 patients presented here refer to the measurements of the nodules one to two weeks after the second-to-last PEI session.

**Figure 3:**
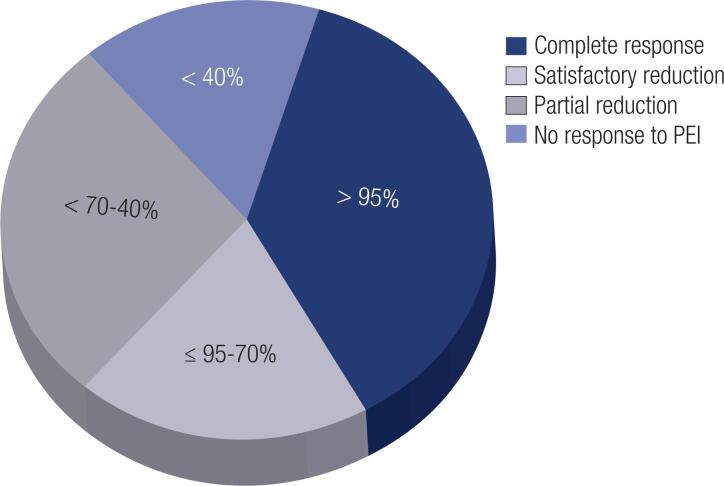
Treated thyroid nodule volume reduction (%).

No response to treatment was considered when the reduction was <40% of the initial volume (Figure 3). Partial reduction was considered when the reduction was ≥40%; at this level, the patient usually reports that the TN has “disappeared”, being able to sleep in lateral decubitus position homolateral to the nodule and/or no longer complaining of obstruction such as coughing and hoarseness. Considering a ≥ 40% volume reduction (no response) as the dependent variable in logistic regression analysis, the PEI reduction power was associated with the number of treatment sessions (p = 0.03, CI [1.1-38.2]) and not with the ultrasonographic characteristics of the nodules and that each PEI session increased the rate of TN reduction by a factor of 6.7 times (p = 0.03).

Pain in the jaw or tooth homolateral to the nodule being treated was a rare self-reported complaint at the time of the injection, that ceased in seconds. Younger patients often said, “it hurts like a puncture”, referring to puncture aspiration of the thyroid, or “it hurts like a tattoo”. A local burning sensation, discomfort, and eventually facial redness were reported. Sometimes, patients reported feeling nothing in one session and pain in another. They classified pain as grade 6 to 7 on the visual analog scale while smiling. Older patients may have complained of pain from the hyperextended neck position necessary during PEI. A patient who presented dysphonia in a PEI session reestablished her voice one week later.

## DISCUSSION

This work compared the degree of reduction in TTNs and UTNs in the same patient, which is considered an ideal comparison model since genetic and environmental patterns were the same for treatment and control. This model to assess this pathology has hitherto not been found in the literature with regard to comparing two usual behaviors in TN monitoring: observation (11,12), which was used for smaller and/or nonfunctioning TNs, and PEI, the technique to be tested. It is a weakness of the study that the volumes of NTs, TTNs and UTNs are not comparable at the beginning, respecting ethical principles. The strong point was having the same observer performing all ultrasounds and a single observer performing all PEI sessions.

This study reported that while some UTNs may have grown, there was a statistically significant reduction in TTNs, similar to the percentages previously reported (13–15). It is possible that, due to the small volume of the UTNs, a slight change (1-2 mm) in the position of the USG probe outside the nodule may have resulted in a measurement bias and a significant increase in the small volume of these UTNs. This possible measurement bias in large TTNs would be insignificant. A similar experimental study with 49 patients undergoing PEI and a control group receiving conservative treatment reported a volume reduction rate of 78.2% in the TTNs, with statistically significant differences between the pre- and postintervention volumes (16). However, the control group showed no statistically significant difference between the pre- and post-observation volumes.

Solid TNs developing over a long period often have gross calcification foci that can obstruct the needle during a PEI session. Immediate repuncture is not prudent, and interruption of the session is recommended to avoid ethanol leakage through the initial puncture. This condition can increase the number of total sessions during treatment. Solid TN should be frequently monitored, and early treatment is indicated once it may result in a substantial reduction with fewer PEI sessions, thereby improving PEI outcomes.

PEI is recommended as a first-line treatment for cystic nodules (8). The comparison between TN reduction by volume and largest diameter showed that the more cystic the nodule was, the greater the response to PEI. Studies exclusively analyzing cysts and/or pseudocysts (9,14,17) reported degrees of reduction greater than or equal to 70%. Better PEI results (fewer sessions) for cystic TNs are due to the aspiration of the liquid inside the cystic or predominantly cystic TNs, which reduces the volume of the nodule. Ethanol avoids liquid recurrence when compared with simple USG-guided fine needle aspiration (FNA) of cystic TNs (8).

There was a more uniform TN reduction response in relation to the number of sessions (Figure 2) considering TN volume rather than the largest diameter. TNs have many different forms, and the formula used to measure spherical structures in different directions, such as TNs, is not ideal. There is a tendency to start alcohol sclerotherapy by infiltrating the areas distant from the carotid, jugular, trachea and esophagus to avoid complications. An oval TN (longer than round) tends to be very close to these structures, slightly reducing its largest diameter and showing less response to PEI when compared to a round TN, which is more uniformly reduced in length, width and depth. The tumor effect is the most important cause of cervical and thoracic compression, which is based on the TN volume and not its diameter. Figure 2 compares TN volume reduction with the number of PEI sessions, showing a positive association between the number of sessions and volume reduction. This relationship was previously described when TN treatment was limited to one or two PEI sessions, which resulted in a lower reduction rate mainly in solid TNs (10).

The results of the present study were underestimated because in 42.7% of cases, the treatment was discontinued, not due to technical criteria but for abandonment. Since the strength of the PEI session to reduce TNs has been proven, it is assumed that if the data from the last session were known, the results obtained in this study would be even better.

Although solid and mixed TNs require more sessions, their response to PEI has been quite satisfactory (10,18,19). In addition, TN reduction occurs even after the end of treatment (20–24). Cystic and predominantly cystic TNs treated with PEI were reported to exhibit a volume reduction of 93% after a seven-year follow-up (23). PEI is safe and effective for treating toxic adenoma and pre-toxic adenoma, and it is widely available in Europe (25).

Additionally, PEI sessions and puncture aspiration have similar costs (23,25). PEI is recognized as an effective low-cost treatment without significant side effects (26).

A lower cutoff point (currently 4 cm) (8) for benign TN would better define treatment, as smaller TNs can be reduced with fewer sessions.

In conclusion, ethanol ablation is a proven method that significantly reduces TN volume and largest diameter both in solid and cystic nodules. It is a well-tolerated procedure, noticeably superior to conservative treatment for all ultrasonographic classifications and can be indicated as a first-line treatment for benign TN.
